# Supplementary material for: Domestication of Chili Pepper Has Altered Fruit Traits Affecting the Oviposition and Feeding Behavior of the Pepper Weevil
Source: Insects. 2021 Jul 12;12(7):630. doi: 10.3390/insects12070630 (PMC8305446; doi:10.3390/insects12070630)
Supplement: Supplementary file 1 [file insects-12-00630-s001.zip › Chabaane e t al _Video S1_Suplementary material.pptx]

## Slide 1
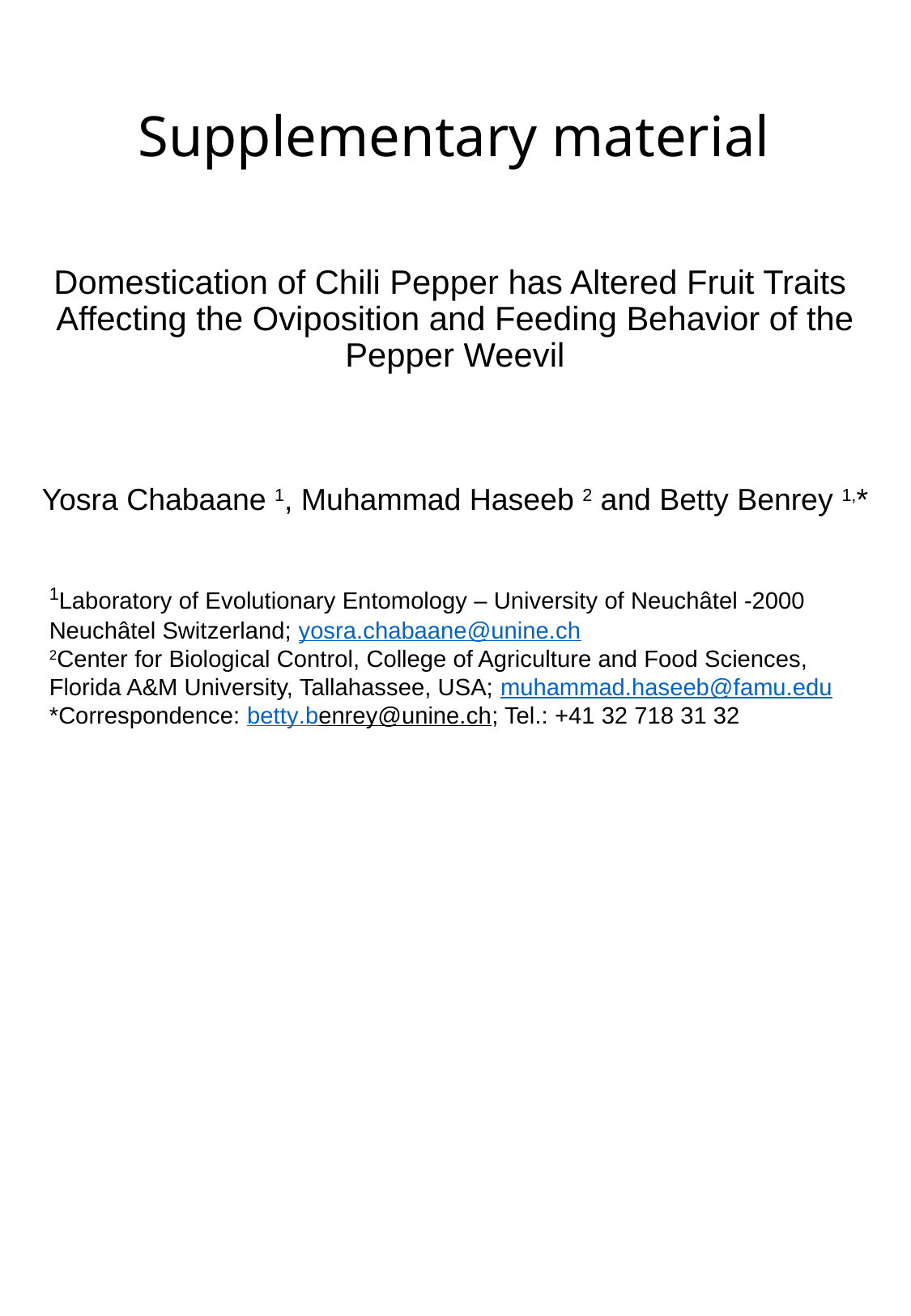

Supplementary material
# Domestication of Chili Pepper has Altered Fruit Traits Affecting the Oviposition and Feeding Behavior of the Pepper Weevil Yosra Chabaane 1, Muhammad Haseeb 2 and Betty Benrey 1,*
1Laboratory of Evolutionary Entomology – University of Neuchâtel -2000 Neuchâtel Switzerland; yosra.chabaane@unine.ch
2Center for Biological Control, College of Agriculture and Food Sciences, Florida A&M University, Tallahassee, USA; muhammad.haseeb@famu.edu
*Correspondence: betty.benrey@unine.ch; Tel.: +41 32 718 31 32
Pupae
Adult
larva

## Slide 2
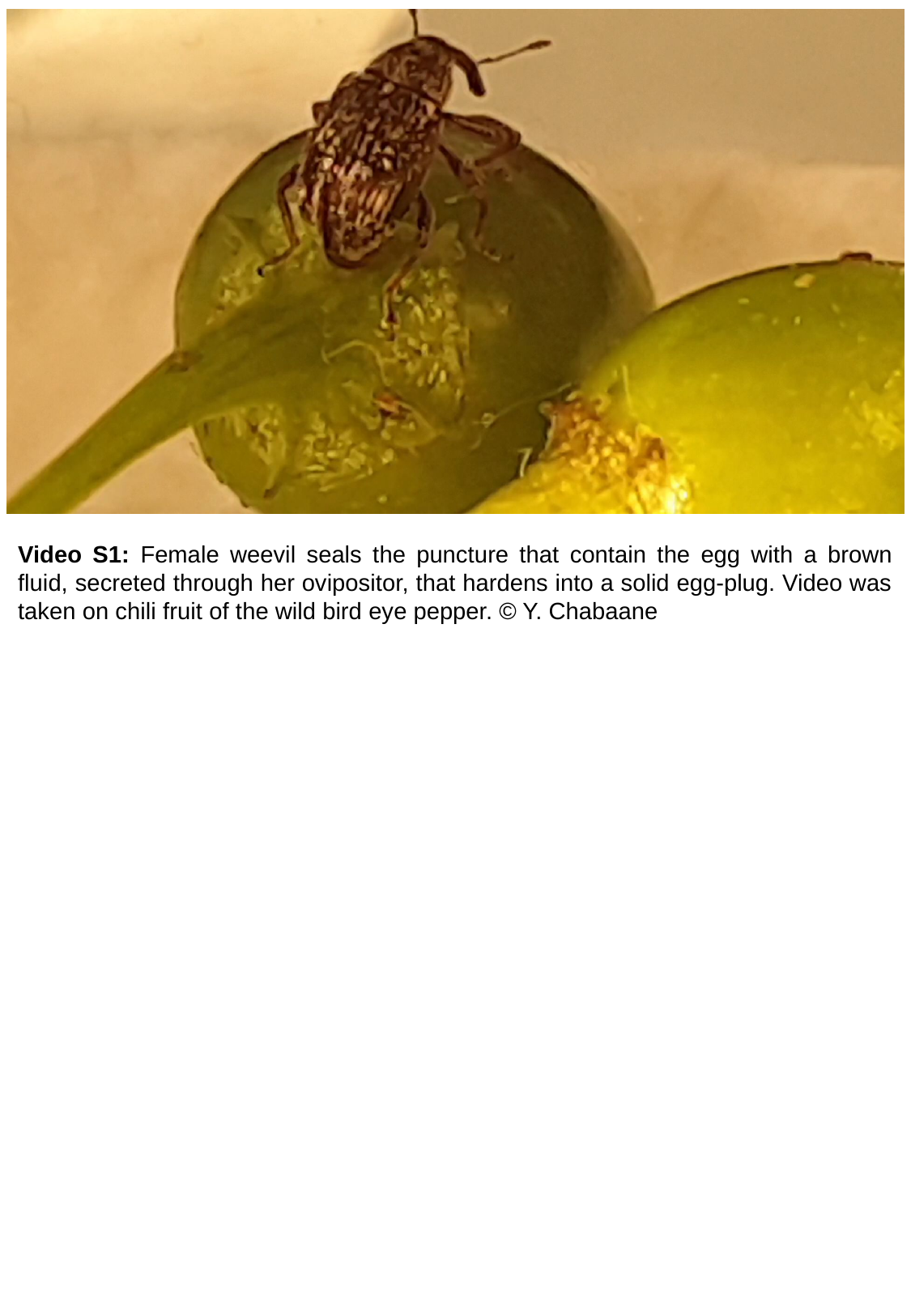

Video S1: Female weevil seals the puncture that contain the egg with a brown fluid, secreted through her ovipositor, that hardens into a solid egg-plug. Video was taken on chili fruit of the wild bird eye pepper. © Y. Chabaane
