# Supplementary material for: Domestication of Chili Pepper Has Altered Fruit Traits Affecting the Oviposition and Feeding Behavior of the Pepper Weevil
Source: Insects. 2021 Jul 12;12(7):630. doi: 10.3390/insects12070630 (PMC8305446; doi:10.3390/insects12070630)
Supplement: Supplementary file 1 [file insects-12-00630-s001.zip › insects-1283004-supplementary.pdf]

# Supplementary material

# DOMESTICATION OF CHILI PEPPER ALTERED FRUIT TRAITS AND AFFECTING THE OVIPOSITION AND FEEDING BEHAVIOR OF THE PEPPER WEEVIL

Yosra Chabaane [1], Muhammed Haseeb [2] and Betty Benrey [1,*]

**1** Laboratory of Evolutionary Entomology – University of Neuchâtel - Switzerland; yosra.chabaane@unine.ch

**2** Center for Biological Control, College of Agriculture and Food Sciences, Florida A&M University, Tallahassee, USA; muhammad.haseeb@famu.edu

*Correspondence: betty.benrey@unine.ch Tel.: +41 32 718 31 32

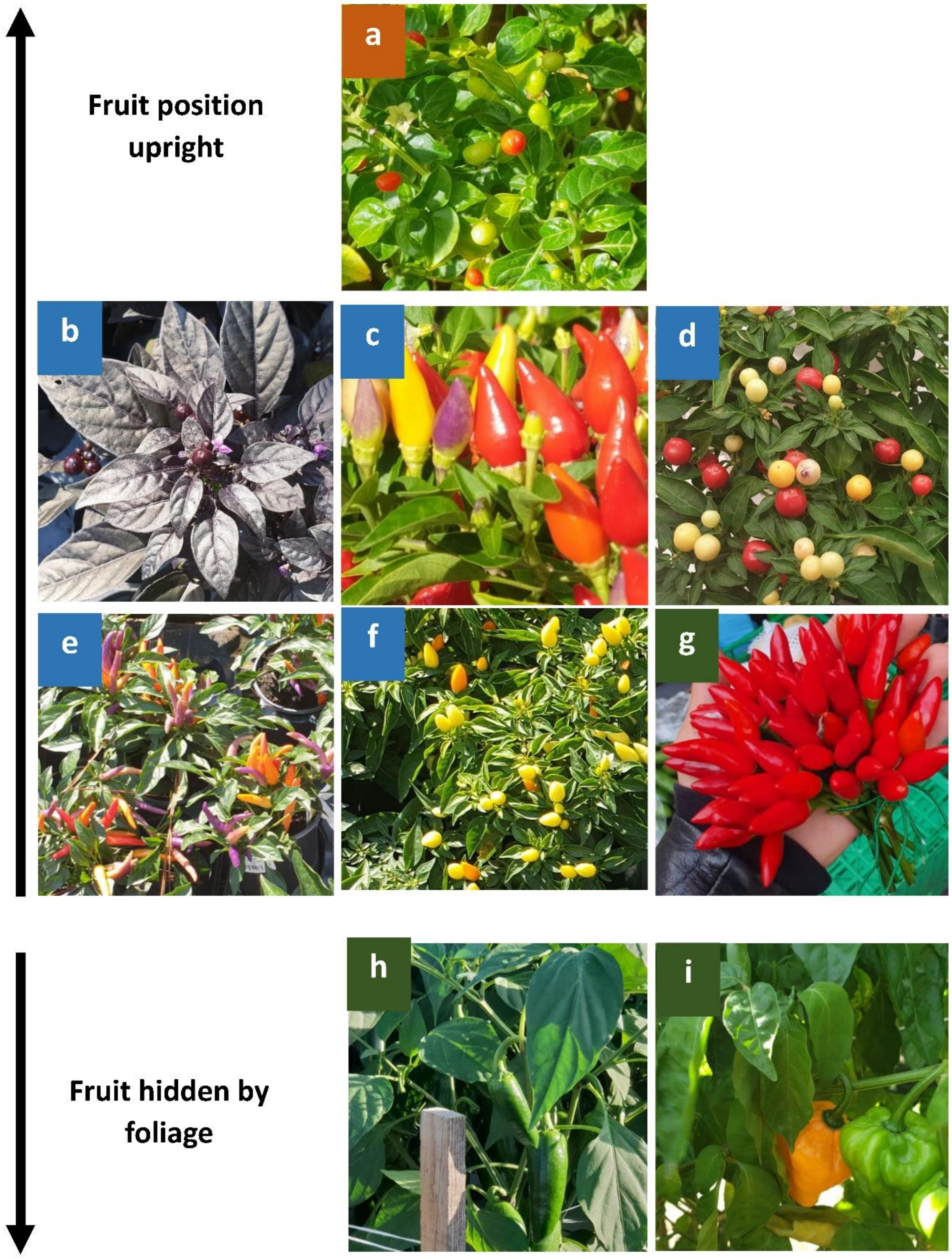


**Figure S1:** Fruit position for wild (Brown), domesticated as ornamental (Blue), and domesticated for consumption (Green) peppers. All images © Y. Chabaane.

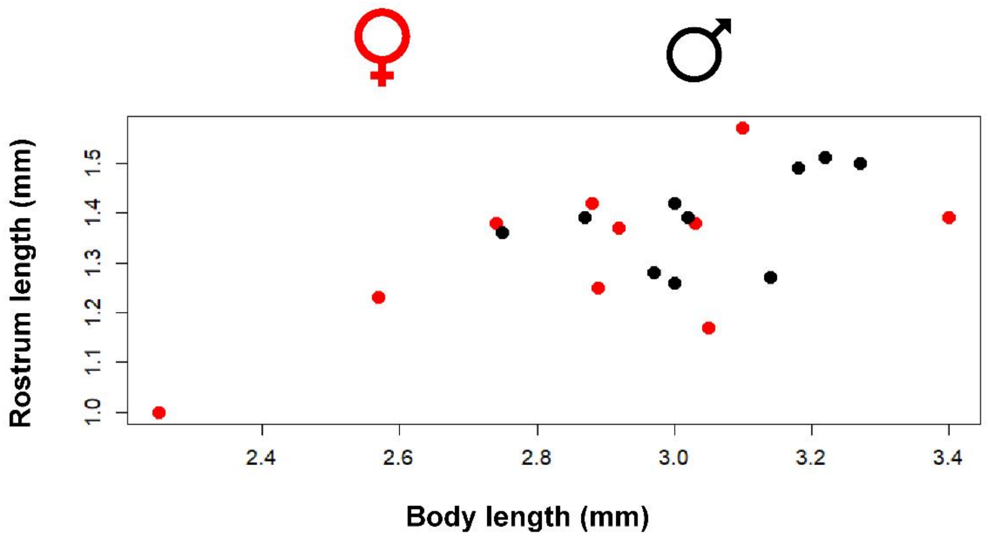


(**a**)

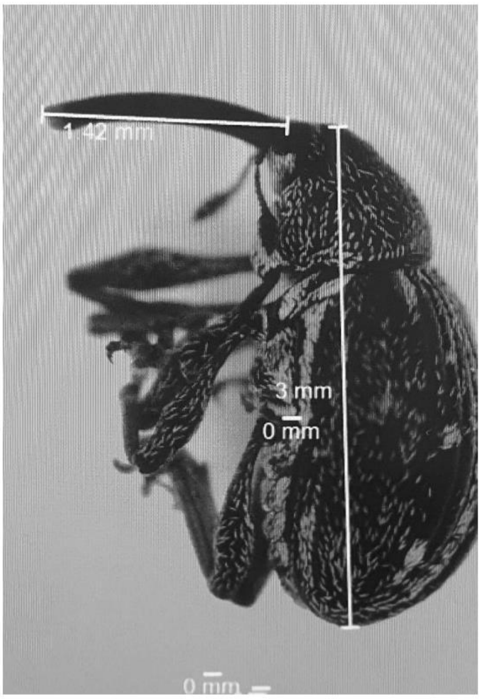


(**b**)

**Figure S2.** (**a**) Correlation between body length (mm) and rostrum length (mm) using 10 males and 10 female weevils. Method used to test correlation = Pearson's product-moment correlation (R = 0.658). (**b**) Measurement of body and rostrum lengths of a female weevil taken using a Leica DMS1000 microscope with build-in 5.0 MPixel CMOS Camera and LAS X Core software (ver. 3.0.13). © Y. Chabaane.
